# Magical thinking in individuals with high polygenic risk for schizophrenia but no non-affective psychoses—a general population study

**DOI:** 10.1038/s41380-022-01581-z

**Published:** 2022-05-03

**Authors:** Aino Saarinen, Leo-Pekka Lyytikäinen, Jarmo Hietala, Henrik Dobewall, Veikka Lavonius, Olli Raitakari, Mika Kähönen, Elina Sormunen, Terho Lehtimäki, Liisa Keltikangas-Järvinen

**Affiliations:** 1 Department of Psychology and Logopedics, University of Helsinki, Helsinki, Finland; 2 Department of Clinical Chemistry, Fimlab Laboratories, and Finnish Cardiovascular Research Center, Tampere, Finland; 3 Department of Cardiology, Heart Center, Tampere University Hospital, Tampere, Finland; 4 Faculty of Medicine and Health Technology, Tampere University, Tampere, Finland; 5 Department of Psychiatry, University of Turku and Turku University Hospital, Turku, Finland; 6 National Institute of Health and Welfare, Helsinki, Finland; 7 Centre for Population Health Research, University of Turku and Turku University Hospital, Turku, Finland; 8 Research Centre of Applied and Preventive Cardiovascular Medicine, University of Turku, Turku, Finland; 9 Department of Clinical Physiology and Nuclear Medicine, Turku University Hospital, Turku, Finland; 10 Department of Clinical Physiology, Tampere University Hospital and Faculty of Medicine and Health Technology, Tampere University, Tampere, Finland

**Keywords:** Genetics, Psychology, Diseases

## Abstract

A strong genetic background for psychoses is well-established. Most individuals with a high genetic risk for schizophrenia, however, do not develop the disorder. We investigated whether individuals, who have a high genetic risk for schizophrenia but no non-affective psychotic disorders, are predisposed to develop milder forms of deviant thinking in terms of magical thinking. Participants came from the population-based Young Finns Study (*n* = 1292). The polygenic risk score for schizophrenia (PRS) was calculated on the basis of the most recent genome-wide association study (GWAS). Psychiatric diagnoses over the lifespan were collected up to 2017 from the registry of hospital care. Magical thinking was evaluated with the Spiritual Acceptance Scale (e.g., beliefs in telepathy, miracles, mystical events, or sixth sense) of the Temperament and Character Inventory in 1997, 2001, and 2012 (participants were 20–50-year-olds). We found that, among those who did not develop non-affective psychotic disorders, high PRS predicted higher magical thinking in adulthood (*p* = 0.001). Further, PRS predicted different developmental courses: a low PRS predicted a steady decrease in magical thinking from age 20 to 50 years, while in individuals with high PRS the decrease in magical thinking ceased in middle age so that their level of magical thinking remained higher than expected for that age. These findings remained when controlling for sex, childhood family environment, and adulthood socioeconomic factors. In conclusion, if high PRS does not lead to a non-affective psychotic disorder, it predicts milder forms of deviant thinking such as elevated magical thinking in adulthood, especially in middle age. The finding enhances our understanding of different outcomes of high genetic psychosis risk.

## Introduction

Familial risk is the strongest single risk factor predicting schizophrenia [1], with offspring of schizophrenia patients having 7.5-fold higher risk for the disorder [2]. Approximately 9–10% of first-degree relatives and 6% of second-degree relatives of schizophrenia patients develop the disorder during their lifetime [3, 4]. Molecular genetic studies first identified a variety of candidate genes for schizophrenia, while most of the candidate-gene findings did not survive in meta-analyses [5]. Recently, polygenic risk scores have been formed on the basis of genome-wide association studies (GWAS), consisting of a comprehensive set of SNPs (i.e., a difference in a single DNA nucleotide) associated with schizophrenia. A major breakthrough occurred in a GWAS study that identified 83 new loci for schizophrenia and reported altogether 128 schizophrenia-related SNPs, including loci associated with genes involved in e.g. glutamatergic neurotransmission, synaptic plasticity, and calcium channels [6]. Thereafter, a meta-analysis including individuals from East-Asian and European ancestries found a total of 208 associations in 176 genetic loci [7]. Most recently, a pre-print (not peer-reviewed) meta-analysis of GWAS studies on schizophrenia in individuals from European and East-Asian ancestries reported common variant associations at 270 distinct loci [8]. There is also evidence for some degree of population-specificity in risk variants for schizophrenia [7]. Recently, it has also been found that most schizophrenia patients may carry ultra-rare coding variants conveying a heightened risk for schizophrenia [9]. Estimates have varied about how much different polygenic risk scores for schizophrenia explain the variation in liability to schizophrenia: some GWAS studies on schizophrenia report estimates of 23–33% [10, 11], whereas a recent review provides an estimate of 7.7% [12].

Previous research has concentrated on individuals with a schizophrenia-susceptible genotype who develop the phenotype (psychosis) during their lifetime. This approach, however, excludes a majority of individuals with schizophrenia-susceptible genotype, because ~90–91% of first-degree relatives of schizophrenia do not develop the disorder during their lifetime [3, 4]. Further, although high values of polygenic risk scores for schizophrenia predict a higher likelihood of psychoses, their specificity in predicting different mental disorders is noted to be comparatively low [6, 13]. Thus, a high polygenic risk may not only represent a liability to psychosis, but also may refer to a broader liability to a variety of harmful outcomes other than psychoses.

Those other outcomes have remained, however, mostly unknown. Within the past few years, a few studies have emerged examining the outcomes of individuals with schizophrenia-susceptible genotype without psychoses. A majority of the studies focused on brain outcomes and found that a high polygenic risk for schizophrenia (PRS) is not associated with structural changes in the brain [14], but correlates with a stronger frontal activity during cognitive tasks [15].

Additionally, there have been some single and conflicting findings on whether PRS relates to cognitive capacity [16, 17] or health behaviors such as smoking, sleep quality, and binge eating [18, 19]. To the best of our knowledge, psychosocial outcomes of polygenic risk for schizophrenia have been examined in a total of five studies. It has been found that high PRS relates to higher trait anxiety [20], higher creativity [21], and a higher number of children in women [22], while PRS is not associated with apathy [23] or the number of days in cohabiting relationships [24].

The present study investigated whether PRS predicts magical thinking, referring to one’s disposition to believe in telepathy, miracles, mystical events, sixth sense, forecasting, or a higher-level force or special power. Thus, magical thinking is an umbrella term including paranormal experiences, mystical ideation, superstitiousness, or anomalous experiences. Most common magical beliefs are found to be precognitive dreams, contact with the dead, or astrological beliefs that occur in 13–15% of the population [25, 26]. According to the current diagnostic classification (the 5th edition of the Diagnostic and Statistical Manual of Mental Disorders, DSM-5) [27], magical thinking can be a sign of various disorders other than psychoses. First, it represents a domain of positive schizotypy, referring to deviant personality development in terms of delusion-like thinking in a restricted domain but a relatively intact level of functioning [27]. Second, if co-occurring with certain other features, it may be a sign of attenuated psychosis syndrome (i.e., milder symptoms below the threshold of psychosis) [27]. In addition, magical thinking can transiently occur in the context of other psychiatric disorders such as obsessive-compulsive disorder or histrionic personality disorder [27]. Taken together, magical thinking can be conceptualized as deviant thinking, typically not distorting reality psychotically but representing milder psychiatric ill-being.

The current study investigated whether individuals, who have a high genetic risk for schizophrenia but no non-affective psychoses, develop stronger deviant thinking, in terms of magical thinking (e.g., beliefs in telepathy, miracles, mystical events, and sixth sense). We used data from the population-based Young Finns Study (YFS), including a population-based sample and a 15-year follow-up of magical thinking. Magical thinking was assessed with an established and well-researched personality inventory. Genetic risk for schizophrenia was assessed in terms of a polygenic risk score for schizophrenia (PRS), calculated on the basis of the most recent GWAS study on schizophrenia that was conducted by the Schizophrenia Working Group of the Psychiatric Genomics Consortium et al. [6].

## Materials and methods

### Participants

The participants come from the Young Finns Study (YFS) which is an ongoing prospective follow-up study. The YFS started in 1980 (baseline measurement) and the participants have been followed over a 37-year prospective follow-up (1983, 1986, 1989, 1992, 1997, 2001, 2007, 2012, and 2017). Participants were selected from the population register of the Social Insurance Institution, and the original sample included 3 596 participants from six different age cohorts (born in 1962, 1965, 1968, 1971, 1974, and 1977). The design of the YFS is described with further details elsewhere [28, 29]. In this study, we included all the participants who had data available on polygenic risk for schizophrenia, psychiatric diagnoses, magical thinking in at least one measurement year (1997, 2001, or 2012), childhood family circumstances (1980/1983), and socioeconomic factors in adulthood (2011). The final sample size of the present study was 1292 participants.

The YFS has been carried out in accordance with the Declaration of Helsinki, and the study design has been approved by the ethical committees of all Finnish Universities with a medical faculty (Universities of Helsinki, Turku, Tampere, Kuopio, and Oulu). All the participants or their parents (if participants aged <18 years) provided informed consent before participation.

The datasets presented in this article are not readily available because YFS is an ongoing follow-up study and the datasets are not anonymised, and the GDPR prevents public sharing of the data. Instead, pseudonymised datasets are possible to share on request, and require a data-sharing agreement between the parties. Requests to access the datasets should be directed to Katri Räikkönen (katri.raikkonen@helsinki.fi) or Niklas Ravaja (niklas.ravaja@helsinki.fi) for childhood psychosocial factors, to Terho Lehtimäki (terho.lehtimaki@tuni.fi) for the genetic dataset, and to Liisa Keltikangas-Järvinen (liisa.keltikangas-jarvinen@helsinki.fi) for magical thinking dataset. The statistical code of the analyses can be requested from the corresponding author.

### Measures

#### Polygenic risk score for schizophrenia

The PRS was calculated on the basis of the summary statistics of the most recent GWAS on schizophrenia that was conducted by Schizophrenia Working Group of the Psychiatric Genomics Consortium et al. and published in Nature [6]. Specifically, a weighted polygenic risk score [30] for every study subject was created by summing up each participant’s schizophrenia-associated risk alleles weighted by risk allele beta estimates [6]. Additionally, an unweighted polygenic risk score was calculated (i.e., summing up schizophrenia-associated risk alleles without weighting them differently). Altogether 128 independent SNPs reaching genome-wide significance in the schizophrenia GWAS were included in the PRS.

More specifically, genotyping was done for 2556 samples using custom build Illumina Human 670k BeadChip at Welcome Trust Sanger Institute. Sample call rate <0.95, excess heterozygosity, sex mismatch, cryptic relatedness (pi-hat >0.2), SNP call rate <0.95, MAF < 0.01, and HWE *p* value <1e-6 were used as quality control filters. After the quality control, there were 2443 samples and 546,677 genotyped SNPs available for further analysis. Genotype Imputation to 1000 Genomes reference was performed using SHAPEIT v1 for haplotype phasing and IMPUTE2 and 1000 Genomes March 2012 haplotypes for genotype imputation. SNPs with imputation information metric >0.3 were considered as well-imputed.

#### Magical thinking

Magical thinking was assessed with the scale of “Spiritual Acceptance” which is a subscale of “Self-Transcendence” in the Temperament and Character Inventory [31]. The subscale includes altogether 13 items that are responded to with a 5-point scale (1 = totally disagree; 5 = totally agree). The scale measures one’s disposition to believe in telepathy, miracles, mystical events, sixth sense, and the existence of a higher-level force. Examples of the items are as follows: “I believe that miracles can happen”; “I think I have a “sixth sense” that tells me what is going to happen”, “I believe that extrasensory perception (e.g., telepathy, forecasting) is really possible”; “I am excited about such life events that cannot be explained scientifically”.

Individuals at clinical high risk for psychosis are shown to have higher scores of Spiritual acceptance [32]. Moreover, high scores of “self-transcendence” are shown to be more common in schizophrenia patients (vs. controls) [33, 34] and schizophrenia patients’ relatives with schizotypal features (vs. controls) [35], to correlate with a schizotypal personality style in a non-clinical sample [36, 37], and to correlate with higher psychotic-like experiences [38, 39]. Additionally, Cloninger’s biopsychosocial model of temperament and character postulates that schizotypal character profile includes high Self-transcendence [40]. Taken together, high Self-transcendence is shown to be closely related to schizotypal features.

For each measurement year (1997, 2001, and 2012), we calculated a mean score of the items for all the participants who had responded to at least 50% of the items of the Spiritual Acceptance scale (at that follow-up point). Of these participants, almost all had responded to all the items. That is, in 1997, 97.4%, 98.2%, and 98.8% of them had no missing values on the scale 1997, 2001, and 2012, respectively. The rest of them had missing values only in one or two items of the scale. In the analyses, we included all the participants who had data available on the mean score of magical thinking in at least one measurement year.

Finally, the mean scores were standardized with the mean and standard deviation of the first measurement year (1997), in order to stabilize the growth-curve trajectories of magical thinking in multilevel models. The scores of magical thinking in 1997–2012 were added as a time-variant outcome variable to the analyses.

#### Psychiatric diagnoses

Participants’ psychiatric diagnoses over their lifespan were collected up to 2017 from the Care Register for Health Care (also known as the Finnish Hospital Discharge Register) (https://thl.fi/en/web/thlfi-en/statistics-and-data/data-and-services/register-descriptions/care-register-for-health-care). In 2017, the participants were 40–55 years old and, thus, older than the typical onset age of schizophrenia [41]. In the register, diagnoses were given in accordance with the diagnostic classification that was prevailing at that time (ICD-8, ICD-9, or ICD-10). ICD-diagnoses were converted to DSM-IV diagnoses, and this conversion is described elsewhere [42]. Diagnoses were grouped into the following categories: [1] non-affective psychotic disorders, [2] substance-related disorders, [3] affective disorders (mood and anxiety disorders), and [4] personality disorders. Participants with many psychiatric diagnoses were categorized into only one of the groups in the following priority order: non-affective psychoses (DSM-IV 295, 297, 298), personality disorders (DSM-IV 301), affective disorders (mood and anxiety disorders, DSM-IV 296, 300, 311), and substance-related disorders (DSM-IV 291, 303, 292, 304, 305).

In this study, we used only data on non-affective psychoses (for excluding participants with non-affective psychoses). The register is found to cover as much as 93% of schizophrenia-spectrum psychoses and 97% of psychotic disorders [43] and has been used also previously for research purposes [44].

#### Socioeconomic covariates in adulthood

Socioeconomic covariates in adulthood (in 2011) included level of income, occupational status, and educational level. The level of income was assessed with a 13-point scale (1 = <5000€; 13 = >60,000€). Occupational status was classified into three categories (1 = manual worker; 2 = lower-grade non-manual worker; 3 = upper-grade non-manual worker). Educational level included three classes (1 = comprehensive school, i.e., the first nine school years; 2 = occupational school or high school; 3 = academic level, i.e., university or college). Each socioeconomic variable was added as a separate time-invariant covariate to the analyses.

#### Childhood psychosocial environment

All the childhood environmental characteristics were assessed with questionnaires presented to the parents in 1980. In case there were missing values in 1980, we imputed them using data from the closest possible follow-up point (in 1983).

The cumulative score of stressful life events included the following factors: change of residence, number of changes of school, parental divorce (whether parents living together or separated), mother’s or father’s death, mother’s or father’s hospitalization within the past 12 months, and child’s hospitalization due to sickness or accident. The cumulative score of adverse socioeconomic circumstances included the following factors: parents’ occupational status, parents’ educational level, family income, unstable employment situation, and overcrowded apartment. The cumulative score of unfavorable emotional family atmosphere included the following factors: emotional distance between the child and parent, parental intolerance toward the child, strict discipline toward the child, parental life dissatisfaction, mother’s or father’s mental disorder, and mother’s or father’s frequent alcohol intoxication. The scales of parental life satisfaction and child-rearing practices have been used previously [45–47]. A detailed description of the assessment of childhood family environment is provided in Supplementary Methods.

### Statistical analyses

First, we excluded all the participants with non-affective psychotic diagnoses (*n* = 74) from the dataset. Next, we examined attrition over the follow-up using independent samples *t* tests (continuous variables) and chi-square tests (categorical variables). The longitudinal associations of PRS with the curve of magical thinking over the 15-year follow-up (1997, 2001, and 2012) were investigated using growth-curve modeling (maximum likelihood estimation) which has stronger statistical power than many other methods [48]. The models estimated the curve of magical thinking for all the participants who had data available in at least one measurement year of magical thinking. The assumptions of the growth-curve models were graphically examined and found to be approximately confirmed. The growth-curve models estimate fixed effects and random effects. Fixed effects can be interpreted as regression coefficients. In Models 1, our greatest interest was the main effect of PRS on the curve of magical thinking. We estimated fixed effects for the PRS, age, age-squared, sex, childhood family circumstances (stressful life events, adverse socioeconomic circumstances, and unfavorable emotional family atmosphere), and socioeconomic factors in adulthood (level of income, occupational status, educational level). In Models 2, we examined whether age modifies the association of PRS with magical thinking and, thus, we added the interaction between PRS and age. In all the models, random effects included individual-level variation in the intercept (i.e., individual-level variance in the mean level of magical thinking over the follow-up) and residual variance (i.e., within-individual variation over the 15-year follow-up). In order to reduce potential multicollinearity in the multilevel models, age was centered on the age of the youngest age cohort in the first measurement year of the outcome variable (i.e., with the age of 20 years as we predicted magical thinking in 1997–2012). Finally, for each growth-curve model, we calculated the Bosker/Snijders pseudo *R*^2^ value which is an established method for multilevel modeling [49, 50]. The Bosked/Snijders pseudo R^2^ is estimated separately for level 1 and level 2 effects. The Bosker/Snijders pseudo *R*^2^ is not very informative in evaluating a single model, but it can provide useful information when comparing various models.

## Results

Included participants, i.e., the YFS participants who were included in the data analyses, were slightly older (42.9 vs. 42.3 years, *p* < 0.01) than dropped-out participants. Women were more likely to participate than men (40.4% vs 31.2%, *p* < 0.001). There was no attrition bias in weighted or unweighted PRS. Included participants had slightly less magical thinking in 1997 (2.66 vs. 2.75, *p* < 0.05) and in 2012 (2.45 vs. 2.58, *p* < 0.01) but not in 2001 (when compared to dropped-out participants). Included participants had on average slightly less-adverse childhood family circumstances: fewer stress-prone events (−0.03 vs. 0.02, *p* < 0.01), less-adverse socioeconomic circumstances (−0.085 vs 0.06, *p* < 0.001), and less-adverse emotional family atmosphere in childhood (−0.03 vs. 0.03, *p* < 0.01) than dropped-out participants. Included participants had also a higher socioeconomic position in adulthood: they had a higher level of income (7.68 vs. 6.76, *p* < 0.001), had less likely a low educational level (2.5% vs. 34.8%, *p* < 0.001), and were more likely upper-level non-manual workers (43.3% vs. 37.2%, *p* < 0.001) (when compared to dropped-out participants).

The descriptive statistics of the study variables among included participants are shown in Table 1. The age range of the participants during the measurement of magical thinking is depicted in Supplementary Fig 1. The measurement years of the study variables are summarized in Supplementary Table 1.

**Table 1:** Descriptive statistics of the study variables (*n* = 1292).

	*M* (SD)	Frequency (%)
Age (in 2012)	42.8 (5.06)	
Sex (Female)		741 (57.4)
*Magical thinking*^a^		
1997	2.67 (0.79)	
2001	2.62 (0.81)	
2012	2.45 (0.80)	
*Polygenic score for schizophrenia*^b^		
Weighted	−0.03 (1.01)	
Unweighted	0.03 (0.99)	
*Educational level in adulthood*		
Comprehensive school		32 (2.5)
Occupational school or high school		680 (52.6)
Academic level		580 (44.9)
*Occupational status in adulthood*		
Manual worker		219 (17.0)
Lower-grade non-manual worker		514 (39.8)
Upper-grade non-manual worker		559 (43.3)
Level of income in adulthood		
Childhood covariates		
Cumulative score for stressful life events	−0.03 (0.39)	
Cumulative score for adverse socioeconomic circumstances	−0.08 (0.62)	
Cumulative score for unfavorable emotional family atmosphere	−0.03 (0.43)	

^a^The unstandardized statistics of magical thinking (in the analyses, we used standardized scores).

^b^The polygenic score for schizophrenia was standardized in the main sample (mean = 0, SD = 1).

### Main analyses

First, we examined possible sex-interactions of the PRS when predicting magical thinking. There were no significant age interactions (*p* = 0.899 and *p* = 0.816 for the interaction of sex with weighted and unweighted PRS, respectively). Consequently, the analyses were run for both sexes in the same analysis.

Then, we examined the main effect of the PRS on the development of magical thinking (in participants without non-affective psychotic diagnoses). The results of the growth-curve models are presented in Tables 2 and 3. Fixed effects can be interpreted as regression coefficients. In Models 1, we found that high weighted PRS (*B* = 0.077, *p* = 0.001, see Table 2) and high unweighted PRS (*B* = 0.082, *p* = 0.001, see Table 3) had a positive main effect on magical thinking (i.e., high weighted and unweighted PRS predicted higher curve of magical thinking in adulthood). The significant main effects of age and age-squared indicated that the curve of magical thinking over age was curvilinear.

**Table 2:** Results of multilevel models with a longitudinal design.

	Magical thinking in adulthood (*n* = 1292)					
	Model 1 pseudo *R*^2^:Level 1:0.114, Level 2:0.120			Model 2 pseudo *R*^2^:Level 1:0.114, Level 2:0.119		
	B	*SE*	*p*	B	*SE*	*p*
*Fixed effects*						
Intercept	−0.029	0.132	0.827	−0.030	0.132	0.820
Age	−0.033	0.004	<0.001	−0.033	0.004	<0.001
Age squared	0.001	0.000	<0.001	0.001	0.000	<0.001
PRS_WGT_	0.077	0.024	0.001	0.036	0.031	0.251
PRS_WGT_ × age				0.003	0.001	0.034
*Random effects*						
Variance of intercept	0.802	0.019	<0.05	0.802	0.019	<0.05
Residual variance	0.488	0.008	<0.05	0.488	0.008	<0.05

Estimates (B) with standard errors (SE) of weighted polygenic risk score for schizophrenia (PRS_WGT_) and age, when predicting standardized scores of magical thinking in adulthood.

“Fixed effects” refer to the classic regression coefficients. “Random effects” refer to the between-individual variation in the intercept and residual variance.

Models 1 and 2 were otherwise identical but, in Model 2, we added the age-interaction of the PRS.

Models were adjusted for sex, childhood family environment (stressful life events, adverse socioeconomic circumstances, unfavorable emotional family atmosphere), and socioeconomic factors in adulthood (level of income, occupational status, educational level).

**Table 3:** Results of multilevel models with a longitudinal design.

	Magical thinking in adulthood (*n* = 1292)					
	Model 1 pseudo *R*^2^: Level 1:0.115, Level 2:0.121			Model 2 pseudo *R*^2^: Level 1:0.115, Level 2:0.120		
	B	*SE*	*p*	B	*SE*	*p*
*Fixed effects*						
Intercept	−0.028	0.132	0.834	−0.029	0.132	0.823
Age	−0.033	0.004	<0.001	−0.033	0.004	<0.001
Age squared	0.001	0.000	<0.001	0.001	0.000	<0.001
PRS_SUM_	0.082	0.024	0.001	0.032	0.031	0.294
PRS_SUM_×age				0.004	0.001	0.011
*Random effects*						
Variance of intercept	0.801	0.019	<0.05	0.802	0.019	<0.05
Residual variance	0.488	0.008	<0.05	0.487	0.008	<0.05

Estimates (B) with standard errors (SE) of unweighted polygenic risk for schizophrenia (PRS_SUM_) and age, when predicting standardized scores of magical thinking in adulthood.

“Fixed effects” refer to the classic regression coefficients. “Random effects” refer to the between-individual variation in the intercept and residual variance.

Models 1 and 2 were otherwise identical but, in Model 2, we added the age-interaction of the PRS.

Models were adjusted for sex, childhood family environment (stressful life events, adverse socioeconomic circumstances, unfavorable emotional family atmosphere), and socioeconomic factors in adulthood (level of income, occupational status, educational level).

**Fig. 1:**
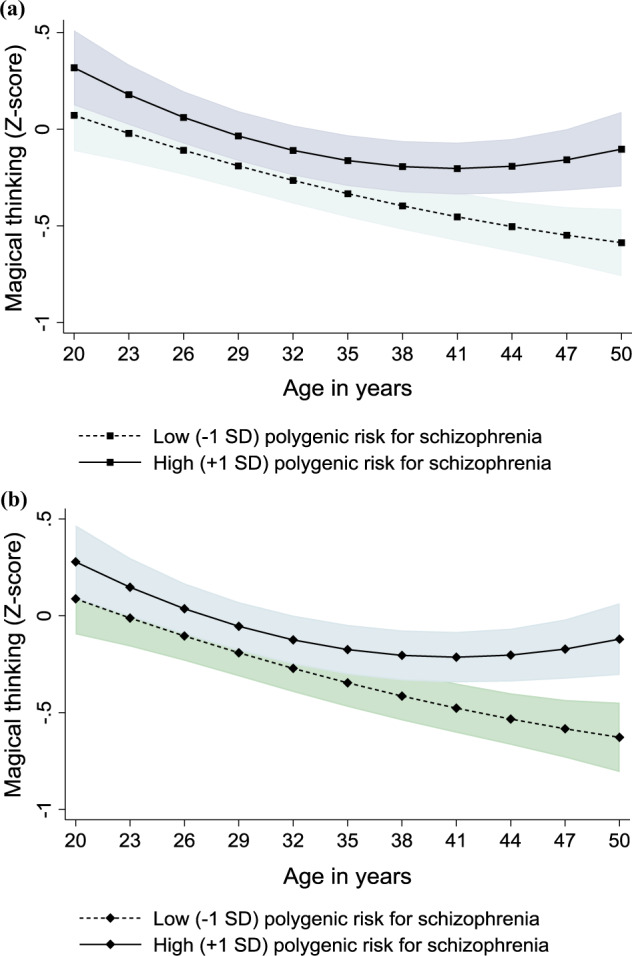
The longitudinal association of the PRS with the curve of magical thinking. Model-predicted values with 95% confidence intervals (marked with gray color) of magical thinking over age separately for participants with low (−1 SD) and high (+1 SD) **a** weighted and **b** unweighted polygenic risk for schizophrenia. Adjusted for sex, childhood family environment (stressful life events, adverse socioeconomic circumstances, unfavorable emotional family atmosphere), and socioeconomic factors in adulthood (level of income, occupational status, educational level).

Second, in Models 2 (Tables 2 and 3), we investigated the interactions between age and the PRS, i.e., whether the association of PRS with magical thinking changes over different age periods. We found a significant positive age-interaction (*p* = 0.034 for weighted PRS; *p* = 0.011 for unweighted PRS), indicating that the associations of the weighted and unweighted PRS with magical thinking were modified by age. The age-interaction is illustrated in Fig. 1. As can be seen from Fig. 1a, b, a low weighted and unweighted PRS predicted a steady decrease in magical thinking from age 20 to 50 years. On the contrary, in individuals with high weighted or unweighted PRS, the decrease in magical thinking seemed to cease in middle age so that their level of magical thinking remained higher than expected for that age.

All these findings (presented in Tables 2 and 3) were adjusted for sex, childhood family environment (stressful life events, adverse socioeconomic circumstances, unfavorable emotional family atmosphere), and socioeconomic factors in adulthood (level of income, occupational status, educational level). Regarding random effects, in Models 1 and 2, we found that there was a significant variance of intercept (i.e., there was individual-level variation in the mean level of magical thinking over the 15-year follow-up). The values of pseudo *R* squares cannot be directly interpreted as percentages of explained variance. Nevertheless, the pseudo *R*^2^ values showed that the weighted and unweighted PRS explained approximately a similar amount of variance.

### Additional analyses

First, as additional analyses, we examined the validity of the PRS. That is, we used logistic regression analysis and predicted the likelihood of non-affective psychoses (0 = not diagnosed with non-affective psychosis; 1=diagnosed with non-affective psychosis) by the weighted and unweighted PRS. We found that weighted PRS (*OR* = 1.585, *p* = 0.005) and unweighted PRS (*OR* = 1.536, *p* = 0.010) predicted higher likelihood of non-affective psychoses. Thus, the validity of the PRS was supported.

Second, we reran the analyses so that also participants with affective disorders (mood/anxiety disorders) were excluded from the sample. This is because the PRS predicts a higher likelihood of non-affective psychoses and also a slightly higher likelihood of affective disorders [51]. The results were replicated. The results are presented in Supplementary Table 2.

Third, as the scale of magical thinking is a subscale of “Self-transcendence” in the TCI, we additionally examined whether the PRS predicts the two other subscales of “Self-transcendence”, i.e., “Self-forgetful experiences” and “Transpersonal identification”. The scale of “Self-forgetful experiences” assesses one’s disposition to be absent-minded, to lose perception of time, place, and surrounding events when being concentrated, and to have sudden experiences of understanding or realization when being relaxed. The scale of “Transpersonal identification” evaluates one’s disposition to experience a strong spiritual or emotional connection to other people, nature, and the universe, disposition to experience that everything seems to be a part of a living organism, and disposition to make effort to protect animals, plants, and the world. A more detailed description of the scales is available in Supplementary Methods. In these additional analyses, we used analogous growth-curve models than in the main analyses. The results are presented in Supplementary Tables 3 and 4. High PRS predicted higher curve of self-forgetful experiences in adulthood (*B* = 0.057, *p* = 0.017 for the weighted PRS; *B* = 0.059, *p* = 0.012 for the unweighted PRS). PRS did not predict transpersonal identification. These findings were adjusted for age, sex, childhood family environment (stressful life events, adverse socioeconomic circumstances, unfavorable emotional family atmosphere), and socioeconomic factors in adulthood (level of income, occupational status, educational level).

## Discussion

The present study showed that individuals, who have a high genetic risk for schizophrenia but no diagnosed non-affective psychoses, may still be predisposed to develop a higher level of magical thinking (i.e., beliefs in telepathy, miracles, mystical events, and sixth sense) in middle age. Further, PRS predicted different developmental courses of this thinking: a low PRS predicted a steady decrease in magical thinking from age 20 to 50 years, while in individuals with high PRS the decrease in magical thinking ceased in middle age. These findings remained when controlling for age, sex, childhood adversities, and socioeconomic position in adulthood. Taken together, we found that if high genetic risk for schizophrenia does not lead to a non-affective psychosis, it may predict milder forms of deviant thinking such as elevated magical thinking in adulthood, especially after young adulthood.

A social marginality hypothesis proposes that socially marginal or disadvantaged groups may develop magical beliefs as a way to take control over their lives and to feel empowerment [52]. Also, it has been speculated that magical thinkers may have experienced childhood adversities [52]. Our results showed that genetic susceptibilities predict magical thinking independently of childhood adversities or a socioeconomic position in adulthood. Magical thinking may develop as a maladaptive coping mechanism or filter that protects from encountering a threatening reality and creates a sense of emotional security [52].

Some previous cross-sectional findings have proposed that magical thinking may relate to an increased risk for psychosis [32, 53]. Additionally, a review stated that high schizotypy (one domain of schizotypy being magical thinking) may play a role in the etiology of schizophrenia-spectrum disorders [54]. Nevertheless, it is necessary to consider that most individuals with high schizotypy will not develop a psychosis [54], not supporting a view that magical thinking could play a clear causal role in the development of psychotic disorders. In addition, to the best of our knowledge, no longitudinal has investigated the temporal relationships between magical thinking and proneness to psychotic disorders. Our findings provide a novel perspective indicating that, in many cases, magical thinking may develop after the typical age at the onset of schizophrenia.

Our additional analyses showed that high PRS may also predict slightly higher proneness to self-forgetful experiences, referring to the disposition to be absent-minded, to lose perception of time, place, and surrounding events when being concentrated, and to have sudden experiences of understanding or realization when being relaxed. This association was comparatively weak but, nevertheless, supported our main finding that PRS may predict mild forms of deviant thinking. Specifically, high self-absorption is related to hallucination proneness [55] and is typically elevated in schizophrenia-spectrum patients [56]. Further, high self-absorption predisposes to intrusive experiences [57], referring to situations where one’s past memories are not consciously processed but are experienced as some kind of non-specific distress in the present moment. Accordingly, it has been emphasized that being “locked in” one’s mind is a central characteristic of the psychosis spectrum [57].

PRS did not, however, predict the trajectory of transpersonal identification in adulthood (i.e., disposition to experience a connection to other people, nature, and the universe; and to experience that everything seems to be a part of a living organism). In mindfulness practices, transpersonal identification is regarded as a beneficial trait that is aimed to be deliberately enhanced: mindfulness aims to enhance connections between the self and others and one’s willingness to benefit from universal well-being beyond the self [58–60]. Transpersonal identification is found to be at a higher level in meditators than in non-meditators [61]. Hence, transpersonal identification seems not to be a maladaptive form of deviant thinking but to represent a “healthy” sort of spirituality.

The onset of schizophrenia typically occurs in early adulthood (at the age of ca. 24–28 years) [41, 62, 63], with another minor peak closer to middle age (among ca. 43–47-year-olds) [41, 62, 63]. Our participants were middle-aged at the time of collecting psychiatric diagnoses from the Finnish Hospital Discharge Register and, thus, not likely to develop psychoses after this study. Nevertheless, we cannot exclude the possibility that some participants may develop a late-onset psychosis despite its rarity. After the age of 65 years, the incidence of schizophrenia is approximately 7.5 per 100,000 person-years [64] and the prevalence of the disorder is ca. 0.1% [65]. A majority of late-onset psychoses are “secondary psychoses” related to dementia, delirium, medications, or other medical conditions [66]. Late-onset schizophrenia most typically includes persecutory delusions or auditory hallucinations [67], indicating that magical thinking is not common in that disorder.

Some limitations must be taken into consideration. First, the correlates of magical thinking, self-transcendence, and spirituality may be partly culturally specific. In Finland, self-transcendence is normatively at a comparatively low level, with high scores correlating with adverse health outcomes such as a higher likelihood of paranoid ideation [68]. In the US self-transcendence is at a higher level than in some European countries [69], and scientific explanations are more common than magical explanations in British but not Mexican individuals’ judgments [70]. Nevertheless, a review proposed that the associations of ST with health or well-being may not be different between cultures [71].

Second, as is common in long-term follow-up studies [72, 73], included participants were more likely to be women, had experienced less-adverse childhood circumstances, and had a higher socioeconomic position in adulthood. However, there was no practical difference in magical thinking or PRS between included and excluded participants, and the data of the Young Finns study has been found to be missing at random [74]. Further, it has been shown that attrition rarely produces any substantial bias in the findings of longitudinal studies [75].

This study had many strengths: we had a fairly large population-based sample, PRS was calculated on the basis of the most recent GWAS study on schizophrenia, and diagnoses were collected from the Finnish national registry of hospital care. Additionally, as magical thinking is found to change slowly over age [76], our 15-year follow-up of personality development provides exceptional possibilities to detect even comparatively minor developmental changes [77].

## Supplementary information

Supplementary Material

## References

[1] Mäki P, Veijola J, Jones PB, Murray GK, Koponen H, Tienari P (2005). Predictors of schizophrenia-a review. Br Med Bull.

[2] Rasic D, Hajek T, Alda M, Uher R (2014). Risk of mental illness in offspring of parents with schizophrenia, bipolar disorder, and major depressive disorder: a meta-analysis of family high-risk studies. Schizophr Bull.

[3] Gottesman II, Laursen TM, Bertelsen A, Mortensen PB (2010). Severe mental disorders in offspring with 2 psychiatrically ill parents. Arch Gen Psychiatry.

[4] Gottesman II. Schizophrenia genesis: the origin of madness. New York: Freeman; 1991.

[5] Johnson EC, Border R, Melroy-Greif WE, de Leeuw CA, Ehringer MA, Keller MC (2017). No evidence that schizophrenia candidate genes are more associated with schizophrenia than noncandidate genes. Biol Psychiatry.

[6] Consortium SWGotPG. (2014). Biological insights from 108 schizophrenia-associated genetic loci. Nature.

[7] Lam M, Chen CY, Li Z, Martin AR, Bryois J, Ma X (2019). Comparative genetic architectures of schizophrenia in East Asian and European populations. Nat Genet.

[8] Consortium TSWGotPG, Ripke S, Walters JTR, O’Donovan MC. Mapping genomic loci prioritises genes and implicates synaptic biology in schizophrenia. MedRxiv. https://www.medrxiv.org/content/10.1101/2020.09.12.20192922v1 2020.

[9] Singh T, Neale BM, Daly MJ. Exome sequencing identifies rare coding variants in 10 genes which confer substantial risk for schizophrenia. MedRxiv. https://www.medrxiv.org/content/10.1101/2020.09.18.20192815v1 2020.

[10] Lee SH, DeCandia TR, Ripke S, Yang J, Sullivan PF, Goddard ME (2012). Estimating the proportion of variation in susceptibility to schizophrenia captured by common SNPs. Nat Genet.

[11] Purcell SM, Wray NR, Stone JL, Visscher PM, O’Donovan MC, Sullivan PF (2009). Common polygenic variation contributes to risk of schizophrenia and bipolar disorder. Nature.

[12] Legge SE, Santoro ML, Periyasamy S, Okewole A, Arsalan A, Kowalec K (2021). Genetic architecture of schizophrenia: a review of major advancements. Psychol Med.

[13] Ripke S, O’Dushlaine C, Chambert K, Moran JL, Kähler AK, Akterin S (2013). Genome-wide association analysis identifies 13 new risk loci for schizophrenia. Nat Genet.

[14] van der Merwe C, Passchier R, Mufford M, Ramesar R, Dalvie S, Stein DJ (2019). Polygenic risk for schizophrenia and associated brain structural changes: a systematic review. Compr Psychiatry.

[15] Dezhina Z, Ranlund S, Kyriakopoulos M, Williams SCR, Dima D (2019). A systematic review of associations between functional MRI activity and polygenic risk for schizophrenia and bipolar disorder. Brain Imaging Behav.

[16] Dimitriadis SI, Lancaster TM, Perry G, Tansey KE, Jones DK, Singh KD, et al. Global brain flexibility during working memory is reduced in a high-genetic-risk group for schizophrenia. Biol Psychiatry Cogn Neurosci Neuroimaging. 2021,16:1176–1184. 10.1016/j.bpsc.2021.01.007 PMC7613444 33524599

[17] Engen MJ, Lyngstad SH, Ueland T, Simonsen CE, Vaskinn A, Smeland O (2020). Polygenic scores for schizophrenia and general cognitive ability: associations with six cognitive domains, premorbid intelligence, and cognitive composite score in individuals with a psychotic disorder and in healthy controls. Transl Psychiatry.

[18] Reed ZE, Jones HJ, Hemani G, Zammit S, Davis OSP (2019). Schizophrenia liability shares common molecular genetic risk factors with sleep duration and nightmares in childhood. Wellcome Open Res.

[19] Wang SH, Lai RY, Lee YC, Su MH, Chen CY, Hsiao PC (2020). Association between polygenic liability for schizophrenia and substance involvement: a nationwide population-based study in Taiwan. Genes Brain Behav.

[20] Hatzimanolis A, Avramopoulos D, Arking DE, Moes A, Bhatnagar P, Lencz T (2018). Stress-dependent association between polygenic risk for schizophrenia and schizotypal traits in young army recruits. Schizophr Bull.

[21] Power RA, Steinberg S, Bjornsdottir G, Rietveld CA, Abdellaoui A, Nivard MM (2015). Polygenic risk scores for schizophrenia and bipolar disorder predict creativity. Nat Neurosci.

[22] Escott-Price V, Pardiñas AF, Santiago E, Walters J, Kirov G, Owen MJ (2019). The relationship between common variant schizophrenia liability and number of offspring in the UK biobank. Am J Psychiatry.

[23] Lyngstad SH, Bettella F, Aminoff SR, Athanasiu L, Andreassen OA, Faerden A (2020). Associations between schizophrenia polygenic risk and apathy in schizophrenia spectrum disorders and healthy controls. Acta Psychiatr Scand.

[24] Hjorthøj C, Uddin MJ, Hougaard DM, Sørensen HJ, Nordentoft M (2019). Polygenic risk for psychiatric disorder and singleness in patients with severe mental illness and controls. J Psychiatr Res.

[25] Dagnall NA, Drinkwater K, Parker A, Clough P. Paranormal experience, belief in the paranormal and anomalous beliefs. Paranthropology. 2016;7:4–15.

[26] Sar V, Alioglu F, Akyuz G (2014). Experiences of possession and paranormal phenomena among women in the general population: are they related to traumatic stress and dissociation?. J Trauma Dissociation.

[27] (APA) APA. Diagnostic and statistical manual of mental disorders, 5th edn 2013.

[28] Raitakari OT, Juonala M, Rönnemaa T, Keltikangas-Järvinen L, Räsänen L, Pietikäinen M (2008). Cohort profile: the cardiovascular risk in Young Finns Study. Int J Epidemiol.

[29] Akerblom HK, Viikari J, Uhari M, Räsänen L, Byckling T, Louhivuori K (1985). Atherosclerosis precursors in Finnish children and adolescents. I. General description of the cross-sectional study of 1980, and an account of the children’s and families’ state of health. Acta Paediatr Scand Suppl.

[30] Igo RP, Kinzy TG, Cooke, Bailey JN (2019). Genetic risk scores. Curr Protoc Hum Genet.

[31] Cloninger CR The Temperament and Character Inventory (TCI): a guide to its development and use. St. Louis, Missouri: Center for Psychobiology of Personality, Washington University 1994.

[32] Mamah D, Cloninger CR, Mutiso VN, Gitonga I, Tele A, Ndetei DM (2020). Personality traits as markers of psychosis risk in kenya: assessment of temperament and character. Schizophr Bull Open.

[33] Smith MJ, Cloninger CR, Harms MP, Csernansky JG (2008). Temperament and character as schizophrenia-related endophenotypes in non-psychotic siblings. Schizophr Res.

[34] Gonzalez-Torres MA, Inchausti L, Ibáñez B, Aristegui M, Fernández-Rivas A, Ruiz E (2009). Temperament and character dimensions in patients with schizophrenia, relatives, and controls. J Nerv Ment Dis.

[35] Bora E, Veznedaroglu B (2007). Temperament and character dimensions of the relatives of schizophrenia patients and controls: the relationship between schizotypal features and personality. Eur Psychiatry.

[36] Laidlaw TM, Dwivedi P, Naito A, Gruzelier JH (2005). Low self-directedness (TCI), mood, schizotypy and hypnotic susceptibility. Personal Individ Differ.

[37] Daneluzzo E, Stratta P, Rossi A (2005). The contribution of temperament and character to schizotypy multidimensionality. Compr Psychiatry.

[38] Prochwicz K, Gawęda Ł (2016). Depression and anxiety mediate the relationship between temperament and character and psychotic-like experiences in healthy subjects. Psychiatry Res.

[39] Nitzburg GC, Malhotra AK, DeRosse P (2014). The relationship between temperament and character and subclinical psychotic-like experiences in healthy adults. Eur Psychiatry.

[40] Cloninger CR, Svrakic DM, Przybeck TR (1993). A psychobiological model of temperament and character. Arch Gen Psychiatry.

[41] Ochoa S, Usall J, Cobo J, Labad X, Kulkarni J (2012). Gender differences in schizophrenia and first-episode psychosis: a comprehensive literature review. Schizophr Res. Treat.

[42] Sormunen E, Saarinen MM, Salokangas RKR, Telama R, Hutri-Kähönen N, Tammelin T (2017). Effects of childhood and adolescence physical activity patterns on psychosis risk-a general population cohort study. NPJ Schizophr.

[43] Sund R (2012). Quality of the Finnish Hospital Discharge Register: a systematic review. Scand J Public Health.

[44] Suvisaari JM, Haukka JK, Tanskanen AJ, Lönnqvist JK (1999). Decline in the incidence of schizophrenia in Finnish cohorts born from 1954 to 1965. Arch Gen Psychiatry.

[45] Hintsa T, Kivimäki M, Elovainio M, Keskivaara P, Hintsanen M, Pulkki-Råback L (2006). Parental socioeconomic position and parental life satisfaction as predictors of job strain in adulthood: 18-year follow-up of the Cardiovascular Risk in Young Finns Study. J Psychosom Res.

[46] Keltikangas-Järvinen L, Pulkki-Råback L, Elovainio M, Raitakari OT, Viikari J, Lehtimäki T (2009). DRD2 C32806T modifies the effect of child-rearing environment on adulthood novelty seeking. Am J Med Genet B Neuropsychiatr Genet.

[47] Makkonen TR, Rönkä T, Timonen S, Valvanne L, Österlund K. Operation family study. Helsinki: Mannerheim League of Child Welfare; 1981.

[48] Gelman A, Hill J. Data analysis using regression and multilevel/hierarchical models. Cambridge: Cambridge University Press; 2006.

[49] Snijders TAB, Bosker R. Multilevel analysis: an introduction to basic and applied multilevel analysis. 2nd edn. London: Sage; 2012.

[50] Snijders TAB, Bosker RJ (1994). Modeled variance in two-level models. Socio Methods Res.

[51] Richards A, Horwood J, Boden J, Kennedy M, Sellers R, Riglin L (2019). Associations between schizophrenia genetic risk, anxiety disorders and manic/hypomanic episode in a longitudinal population cohort study. Br J Psychiatry.

[52] Irwin HJ. The psychology of paranormal belief: A researcher’s handbook: Univ of Hertfordshire Press; 2009.

[53] Kwapil TR, Miller MB, Zinser MC, Chapman J, Chapman LJ (1997). Magical ideation and social anhedonia as predictors of psychosis proneness: a partial replication. J Abnorm Psychol.

[54] Barrantes-Vidal N, Grant P, Kwapil TR (2015). The role of schizotypy in the study of the etiology of schizophrenia spectrum disorders. Schizophr Bull.

[55] Perona-Garcelán S, García-Montes JM, Rodríguez-Testal JF, Ruiz-Veguilla M, Benítez-Hernández Mdel M, López-Jiménez AM (2013). Relationship of absorption, depersonalisation, and self-focused attention in subjects with and without hallucination proneness. Cogn Neuropsychiatry.

[56] Kállai J, Vincze G, Török IA, Hargitai R, Rózsa S, Hartung I, et al. Cognitive gain or handicap magical ideation and self-absorption in a clinical and non-clinical sample. Front Psychol 2021;12:613074. 10.3389/fpsyg.2021.613074 PMC7952430 33716876

[57] Sellers R, Wells A, Morrison AP (2018). Are experiences of psychosis associated with unhelpful metacognitive coping strategies? A systematic review of the evidence. Clin Psychol Psychother.

[58] Kang Y (2019). Examining interpersonal self-transcendence as a potential mechanism linking meditation and social outcomes. Curr Opin Psychol.

[59] Verhaeghen P (2019). The mindfulness manifold: exploring how self-preoccupation, self-compassion, and self-transcendence translate mindfulness into positive psychological outcomes. Mindfulness.

[60] Vago DR, Silbersweig DA (2012). Self-awareness, self-regulation, and self-transcendence (S-ART): a framework for understanding the neurobiological mechanisms of mindfulness. Front Hum Neurosci.

[61] Majeski RA. The relationship of transpersonal self-transcendence, extraversion, openness to experience, and psychological well-being in mature adult female meditators and non-meditators. University of Maryland, College Park, 1998.

[62] Häfner H, Hambrecht M, Löffler W, Munk-Jørgensen P, Riecher-Rössler A (1998). Is schizophrenia a disorder of all ages? A comparison of first episodes and early course across the life-cycle. Psychol Med.

[63] Chen L, Selvendra A, Stewart A, Castle D (2018). Risk factors in early and late onset schizophrenia. Compr Psychiatry.

[64] Stafford J, Howard R, Kirkbride JB (2018). The incidence of very late-onset psychotic disorders: a systematic review and meta-analysis, 1960-2016. Psychol Med.

[65] Copeland JR, Dewey ME, Scott A, Gilmore C, Larkin BA, Cleave N (1998). Schizophrenia and delusional disorder in older age: community prevalence, incidence, comorbidity, and outcome. Schizophr Bull.

[66] Tampi RR, Young J, Hoq R, Resnick K, Tampi DJ (2019). Psychotic disorders in late life: a narrative review. Ther Adv Psychopharmacol.

[67] McClure FS, Gladsjo JA, Jeste DV (1999). Late-onset psychosis: clinical, research, and ethical considerations. Am J Psychiatry.

[68] Saarinen A, Rosenström T, Hintsanen M, Hakulinen C, Pulkki-Råback L, Lehtimäki T (2018). Longitudinal associations of temperament and character with paranoid ideation: a population-based study. Psychiatry Res.

[69] Farmer A, Mahmood A, Redman K, Harris T, Sadler S, McGuffin P (2003). A sib-pair study of the temperament and character inventory scales in major depression. Arch Gen Psychiatry.

[70] Subbotsky E, Quinteros G (2002). Do cultural factors affect causal beliefs? Rational and magical thinking in Britain and Mexico. Br J Psychol.

[71] Garcia-Romeu A (2010). Self-transcendence as a measurable transpersonal construct. J Transpers Psychol.

[72] de Graaf R, Bijl RV, Smit F, Ravelli A, Vollebergh WA (2000). Psychiatric and sociodemographic predictors of attrition in a longitudinal study: The Netherlands Mental Health Survey and Incidence Study (NEMESIS). Am J Epidemiol.

[73] Tambs K, Rønning T, Prescott CA, Kendler KS, Reichborn-Kjennerud T, Torgersen S (2009). The Norwegian Institute of Public Health twin study of mental health: examining recruitment and attrition bias. Twin Res. Hum Genet.

[74] Pulkki-Råback L, Elovainio M, Hakulinen C, Lipsanen J, Hintsanen M, Jokela M (2015). Cumulative effect of psychosocial factors in youth on ideal cardiovascular health in adulthood: the Cardiovascular Risk in Young Finns Study. Circulation.

[75] Saiepour N, Najman JM, Ware R, Baker P, Clavarino AM, Williams GM (2019). Does attrition affect estimates of association: a longitudinal study. J Psychiatr Res.

[76] Ferchiou A, Todorov L, Lajnef M, Baudin G, Pignon B, Richard JR,. et al. [Schizotypal Personality Questionnaire-Brief - Likert format: Factor structure analysis in general population in France]. Encephale. 2017;43:558–63. 10.1016/j.encep.2016.05.011 27644915

[77] Josefsson K, Jokela M, Cloninger CR, Hintsanen M, Salo J, Hintsa T (2013). Maturity and change in personality: developmental trends of temperament and character in adulthood. Dev Psychopathol.

