# Supplementary material for: Magical thinking in individuals with high polygenic risk for schizophrenia but no non-affective psychoses—a general population study
Source: Mol Psychiatry. 2022 May 3;27(8):3286–93. doi: 10.1038/s41380-022-01581-z (PMC9708578; doi:10.1038/s41380-022-01581-z)
Supplement: Supplementary file 1 — Supplementary Material [file 41380_2022_1581_MOESM1_ESM.docx]

**Supplementary Methods.** Assessment of childhood psychosocial environment.

Childhood family environment was assessed with **three cumulative scores: (1) stress-prone childhood events, (2) disadvantageous emotional childhood environment, and (3) unfavorable socioeconomic childhood environment.** All the childhood environmental characteristics were assessed with questionnaires presented for the parents in 1980. In case there were missing values in 1980, we imputed them using data from the closest possible follow-up point (in 1983).

**Stress-prone childhood events.** The cumulative score of stress-prone events included the following factors: change of residence, number of change of school, parental divorce (whether parents living together or separated), mother’s or father’s death, mother’s or father’s hospitalization within the past 12 months (number of days in hospital, ranging from “1 = no days” to “5 = more than 30 days”), and child’s hospitalization due to sickness or accident (no/yes). Each item was standardized by age cohort (i.e., *M* = 0, *SD* = 1 within each age cohort), and we calculated a mean score of the standardized items (each item was weighted similarly).

**Unfavorable socioeconomic childhood environment.** The cumulative score of socioeconomic family environment included the following factors: parents’ occupational status (1 = upper-grade non-manual worker, 2 = lower-grade non-manual worker, 3 = manual worker manual worker), parents’ educational level (1 = academic level, 2 = high school or occupational school, 3 = comprehensive school), family income (1 = more than 100 000 Finnish mark, 8 = less than 20 000 Finnish mark), unstable employment situation (1 = at least one parent was unemployed or in a long-term sick leave, 0 = other employment situations), and over-crowded apartment (family size in relation to number of rooms at home, with higher values referring to higher over-crowding). Each item was standardized by age cohort (i.e., *M* = 0, *SD* = 1 within each age cohort), and we calculated a mean score of the standardized items (each item was weighted similarly).

**Disadvantageous emotional childhood environment.** The cumulative score of disadvantageous emotional childhood environment included the following factors: emotional distance between the child and parent, parental intolerance toward the child, strict discipline toward the child, parental life dissatisfaction, mother’s or father’s mental disorder (no/yes), and mother’s or father’s frequent alcohol intoxication (ranging from “1=never” to “8=daily”). Each item was standardized by age cohort (i.e., *M* = 0, *SD* = 1 within each age cohort), and we calculated a mean score of the standardized items (each item was weighted similarly).

*Emotional distance between the parent and child* was evaluated with a four-item questionnaire (e.g., “The child is emotionally important for me”, ”I can realize myself with the child”). The items were responded with a 5-point scale (e.g., 1 = little, 5 = much). In this study, each item was first standardized within the age cohort (*M* = 0, *SD* = 1), then a total score of the items was calculated, and the total score was standardized with the age cohort (*M* = 0, *SD* = 1). *Parental intolerance toward the child* was evaluated with a three-item scale (“I get nervous when spending time with the child”, “The child is a burden in challenging situations”, “The child consumes my time too much”). The items were responded with a 5-point scale (1 = frequently, 5 = never). *Strict discipline toward the child* was measured with a three-item scale (“Disciplinary actions are often needed at home due to child’s aggressiveness”; “Disciplinary actions do not affect the child enough”; “Disciplinary actions are necessary in the rearing of the child”). In this study, each item was first standardized within the age cohort (*M* = 0, *SD* = 1), then a total score of the items was calculated, and the total score was standardized with the age cohort (*M* = 0, *SD* = 1). The items measuring parenting have been used also previously (Keltikangas-Järvinen et al., 2009; Pulkki, Keltikangas-Järvinen, Ravaja, & Viikari, 2003).

*Parental life satisfaction* was assessed with a three-item questionnaire measuring parent’s satisfaction in three life sectors: as a parent, spouse, and employee. The items were responded with a 5-point scale (1 = satisfied, 5 = dissatisfied). This questionnaire has been adapted from the Operation Family Study questionnaire (Makkonen, Rönkä, Timonen, Valvanne, & Österlund, 1981) and has been used also previously (Hintsa et al., 2006; Saarinen et al., 2021). In this study, each item was first standardized within the age cohort (*M* = 0, *SD* = 1), then a total score of the items was calculated, and the total score was standardized with the age cohort (*M* = 0, *SD* = 1).

**“Self-forgetful experiences” and “Transpersonal identification”.** The scales of “Self-forgetful experiences (vs. Self-conscious experience)” and “Transpersonal identification (vs. Self-differentiation)” are subscales of “Self-Transcendence” in the Temperament and Character Inventory (Cloninger, 1994). The scale of “Self-forgetful experiences” includes 11 items (e.g., “Often when I am concentrated on something, I lose my perception of time”; ”I am often said to be absent-minded because I get absorbed in my doing so that I cannot notice anything else anymore”; ”Other people often think that I am in some other world because I am so totally unaware of things happening around me”). The scale of “Transpersonal Identification includes 9 items (e.g., “I often feel having a connection with other people as if there was no difference between us at all”; “I often make an effort to protect animals and plants from dying out”; ”I often feel having a connection with the nature as if everything was a part of a living organism”). The items were responded with a 5-point scale (1 = totally disagree; 5 = totally agree).

For each measurement year (1997, 2001, and 2012), we calculated a mean score of the scale items for all the participants who had responded to at least 50 % of the items. Finally, the mean scores were standardized with the mean and standard deviation of the first measurement year (1997), in order to stabilize the growth curve trajectories of “Self-forgetful experiences” and “Transpersonal identification” in multilevel models. The variables of “Self-forgetful experiences” and “Transpersonal identification” were added as time-variant predictors to the analyses.

|  |  |  |  |  | The cohort born in 1962 (*n* = 217) | | | | | |
| --- | --- | --- | --- | --- | --- | --- | --- | --- | --- | --- |
|  |  |  |  | The cohort born in 1965 (*n* = 245) | | | | | |  |
|  |  |  | The cohort born in 1968 (*n* = 219) | | | | | |  |  |
|  |  | The cohort born in 1971 (*n* = 210) | | | | | |  |  |  |
|  | The cohort born in 1974 (*n* = 209) | | | | | |  |  |  |  |
| The cohort born in 1977 (*n* = 192) | | | | | |  |  |  |  |  |
| **20** | **23** | **26** | **29** | **32** | **35** | **38** | **41** | **44** | **47** | **50** |
| **Age in years over the follow-up from 1997 to 2012** | | | | | | | | | | |

**Supplementary Figure 1.** An illustration of the age of each cohort during the follow-up of magical thinking.

**Supplementary Table 1.** The measurement years of the study variables (participants’ age range within the parentheses).

|  | **1980**  **(3‒18 years)** | **1983**  **(6‒21 years)** | **1997**  **(20‒35 years)** | **2001**  **(24‒39 years)** | **2011/2012**  **(34‒50 years)** | **2021**  **(age not relevant)** |
| --- | --- | --- | --- | --- | --- | --- |
| Cumulative childhood scores |  |  |  |  |  |  |
| Stressful life events | X | X |  |  |  |  |
| Socioeconomic circumstances | X | X |  |  |  |  |
| Emotional family atmosphere | X | X |  |  |  |  |
| Adulthood socioeconomic factors |  |  |  |  |  |  |
| Educational level |  |  |  |  | X |  |
| Occupational status |  |  |  |  | X |  |
| Level of income |  |  |  |  | X |  |
| Magical thinking |  |  | X | X | X |  |
| Calculation of the PRS |  |  |  |  |  | X |

**Supplementary Table 2.** Results of multilevel models with longitudinal design. Estimates (B) with standard errors (SE) of polygenic risk for schizophrenia and age, when predicting standardized scores of magical thinking in adulthood. *Note:* participants with mood/anxiety disorders were excluded.

|  | Magical thinking in adulthood (*n* = 1261) | | | | | | |
| --- | --- | --- | --- | --- | --- | --- | --- |
|  | Weighted PRS  pseudo R^2^:  Level 1: 0.114, Level 2: 0.120 | | |  | Unweighted PRS  pseudo R^2^:  Level 1: 0.115, Level 2: 0.121 | | |
|  | B | *SE* | *p* |  | B | *SE* | *p* |
| Fixed effects |  |  |  |  |  |  |  |
| Intercept | -0.063 | 0.134 | 0.635 |  | -0.061 | 0.134 | 0.645 |
| Age | -0.033 | 0.005 | < 0.001 |  | -0.033 | 0.005 | < 0.001 |
| Age squared | 0.006 | 0.000 | < 0.001 |  | 0.006 | 0.000 | < 0.001 |
| PRS_WGT_ | 0.078 | 0.024 | 0.001 |  |  |  |  |
| PRS_SUM_ |  |  |  |  | 0.082 | 0.024 | 0.001 |
|  |  |  |  |  |  |  |  |
| Random effects |  |  |  |  |  |  |  |
| Variance of intercept | 0.798 | 0.019 | < 0.05 |  | 0.797 | 0.019 | < 0.05 |
| Residual variance | 0.489 | 0.008 | < 0.05 |  | 0.489 | 0.008 | < 0.05 |
| *Note*: “Fixed effects” refer to the classic regression coefficients. “Random effects” refer to between-individual variation in the intercept and residual variance.  Models were adjusted for sex, childhood family environment (stressful life events, adverse socioeconomic circumstances, unfavorable emotional family atmosphere), and socioeconomic factors in adulthood (level of income, occupational status, educational level). | | | | | | | |

**Supplementary Table 3.** Results of multilevel models with longitudinal design. Estimates (B) with standard errors (SE) of polygenic risk for schizophrenia and age, when predicting standardized scores of self-forgetful experiences in adulthood.

|  | Self-forgetful experiences in adulthood (*n* = 1292) | | | | | | |
| --- | --- | --- | --- | --- | --- | --- | --- |
|  | Weighted PRS  pseudo R^2^:  Level 1: 0.039, Level 2: 0.027 | | |  | Unweighted PRS  pseudo R^2^:  Level 1: 0.039, Level 2: 0.028 | | |
|  | B | *SE* | *p* |  | B | *SE* | *p* |
| Fixed effects |  |  |  |  |  |  |  |
| Intercept | 0.073 | 0.131 | 0.580 |  | 0.073 | 0.131 | 0.576 |
| Age | -0.048 | 0.005 | < 0.001 |  | -0.048 | 0.005 | < 0.001 |
| Age squared | 0.001 | 0.000 | < 0.001 |  | 0.001 | 0.000 | < 0.001 |
| PRS_WGT_ | 0.057 | 0.023 | 0.017 |  |  |  |  |
| PRS_SUM_ |  |  |  |  | 0.059 | 0.023 | 0.012 |
|  |  |  |  |  |  |  |  |
| Random effects |  |  |  |  |  |  |  |
| Variance of intercept | 0.762 | 0.019 | < 0.05 |  | 0.762 | 0.019 | < 0.05 |
| Residual variance | 0.570 | 0.009 | < 0.05 |  | 0.570 | 0.009 | < 0.05 |
| *Note*: “Fixed effects” refer to the classic regression coefficients. “Random effects” refer to between-individual variation in the intercept and residual variance.  Models were adjusted for sex, childhood family environment (stressful life events, adverse socioeconomic circumstances, unfavorable emotional family atmosphere), and socioeconomic factors in adulthood (level of income, occupational status, educational level). | | | | | | | |

**Supplementary Table 4.** Results of multilevel models with longitudinal design. Estimates (B) with standard errors (SE) of polygenic risk for schizophrenia and age, when predicting standardized scores of transpersonal identification in adulthood.

|  | Transpersonal identification in adulthood | | | | | | |
| --- | --- | --- | --- | --- | --- | --- | --- |
|  | Weighted PRS  pseudo R^2^:  Level 1: 0.044, Level 2: 0.015 | | |  | Unweighted PRS  pseudo R^2^:  Level 1: 0.044, Level 2: 0.016 | | |
|  | B | *SE* | *p* |  | B | *SE* | *p* |
| Fixed effects |  |  |  |  |  |  |  |
| Intercept | 0.609 | 0.135 | < 0.001 |  | 0.610 | 0.135 | < 0.001 |
| Age | -0.054 | 0.005 | < 0.001 |  | -0.054 | 0.005 | < 0.001 |
| Age squared | 0.001 | 0.000 | < 0.001 |  | 0.001 | 0.000 | < 0.001 |
| PRS_WGT_ | 0.028 | 0.024 | 0.254 |  |  |  |  |
| PRS_WGT_*Age |  |  |  |  | 0.033 | 0.024 | 0.171 |
|  |  |  |  |  |  |  |  |
| Random effects |  |  |  |  |  |  |  |
| Variance of intercept | 0.785 | 0.020 | < 0.05 |  | 0.785 | 0.020 | < 0.05 |
| Residual variance | 0.587 | 0.010 | < 0.05 |  | 0.587 | 0.010 | < 0.05 |
| *Note*: “Fixed effects” refer to the classic regression coefficients. “Random effects” refer to between-individual variation in the intercept and residual variance.  Models were adjusted for sex, childhood family environment (stressful life events, adverse socioeconomic circumstances, unfavorable emotional family atmosphere), and socioeconomic factors in adulthood (level of income, occupational status, educational level). | | | | | | | |

**Supplementary References.**

Hintsa, T., Kivimäki, M., Elovainio, M., Keskivaara, P., Hintsanen, M., Pulkki-Råback, L., & Keltikangas-Järvinen, L. (2006). Parental socioeconomic position and parental life satisfaction as predictors of job strain in adulthood: 18-year follow-up of the Cardiovascular Risk in Young Finns Study. J Psychosom Res, 61(2), 243-249. doi:10.1016/j.jpsychores.2006.05.014

Keltikangas-Järvinen, L., Pulkki-Råback, L., Elovainio, M., Raitakari, O. T., Viikari, J., & Lehtimäki, T. (2009). DRD2 C32806T modifies the effect of child-rearing environment on adulthood novelty seeking. Am J Med Genet B Neuropsychiatr Genet, 150b(3), 389-394. doi:10.1002/ajmg.b.30830

Makkonen, T. R., I., Rönkä, T., Timonen, S., Valvanne, L., & Österlund, K. (1981). Operation family study. Helsinki: Mannerheim League of Child Welfare.

Pulkki, L., Keltikangas-Järvinen, L., Ravaja, N., & Viikari, J. (2003). Child-rearing attitudes and cardiovascular risk among children: moderating influence of parental socioeconomic status. Prev Med, 36(1), 55-63. doi:10.1006/pmed.2002.1125

Saarinen, A., Keltikangas-Järvinen, L., Dobewall, H., Ahola-Olli, A., Salmi, M., Lehtimäki, T., . . . Hintsanen, M. (2021). Risky emotional family environment in childhood and depression-related cytokines in adulthood: The protective role of compassion. Dev Psychobiol. doi:10.1002/dev.22070
